# Photobiomodulation and platelet‐rich fibrin in the gastrocnemius muscle submitted to calcaneal tendinopathy in rats

**DOI:** 10.1111/php.70034

**Published:** 2025-09-10

**Authors:** Pâmela Andressa Pauletto, Caroline Hammerschmitt Eduardo Schmitt, Fransael Franklyn Araújo da Silva, Maria Eduarda Luckner, Lucinéia de Fátima Chasko Ribeiro, Márcia Miranda Torrejais, Gladson Ricardo Flor Bertolini

**Affiliations:** ^1^ Universidade Estadual Do Oeste Do Paraná (UNIOESTE) Cascavel Brazil; ^2^ Universidade Federal do Paraná (UFPR) Palotina Brazil

**Keywords:** gastrocnemius muscle, low‐intensity light therapy, muscle atrophy, platelet‐rich fibrin, tendinopathy, therapeutic

## Abstract

The study investigated the impact of different treatments on recovery from calcaneal tendinopathy in rats, focusing on the gastrocnemius muscle. Tendinopathy is caused by repetitive overload, leading to structural collagen damage and chronic muscle inflammation. Three therapeutic approaches were compared: photobiomodulation (PBM), advanced platelet‐rich fibrin (A‐PRF) injection, and a combination of the two. Seventy‐five rats were separated into five groups: control (CON), injury (LES), PRF (A‐PRF), photobiomodulation (PBM) and combined therapy (A‐PRF + PBM). Tendinopathy was induced by compression of the calcaneal tendon. The treatment was applied at specific intervals, and the animals were assessed for muscle strength and subjected to histological and morphometric analysis of the muscle. LES showed the lowest muscle strength. The treated groups (PBM, A‐PRF and A‐PRF + PBM) had an increase in strength between days 7 and 21, but there were no signs of muscle damage or significant recovery in the muscle fibers. The combined therapy group showed greater production of type III collagen in the connective tissue, indicating a more significant repair effect. In contrast, the neuromuscular junctions in the groups treated with PRF were smaller, suggesting possible structural alterations. The combination of therapies showed superior results to treatment alone, promoting greater tissue repair in the gastrocnemius muscle, especially due to the increase in type III collagen.

## Graphical abstract

The study evaluated different treatments for calcaneal tendinopathy in rats, focusing on the gastrocnemius muscle. Photobiomodulation (PBM), advanced platelet‐rich fibrin (A‐PRF), and their combination were tested. Treated animals showed improved muscle strength between 7 and 21 days, but without significant recovery of muscle fibers. The combined therapy promoted greater type III collagen production, indicating more effective repair, while PRF alone showed changes in neuromuscular junctions. Therefore, the combination of PBM and A‐PRF demonstrated superior results, enhancing tissue repair.

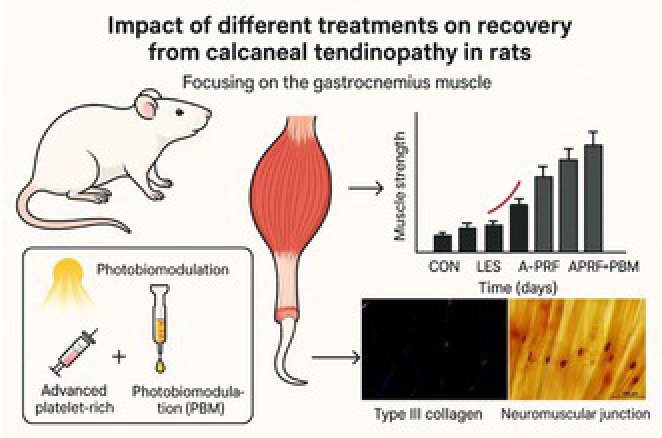

**Abbreviations**
A‐PRF: advanced platelet‐rich fibrin
A‐PRF+PBM: group combined therapy
CON: control group
EGF: epidermal growth factor
FGF: fibroblast growth factor
IGF‐1: insulin‐like growth factor type 1
IL‐10: Interleukin‐10
IL‐1β: Interleukin‐1β
IL‐6: Interleukin‐6
LSD: least significant difference
NMJs: neuromuscular junctions
PBM: photobiomodulation
PDGF: platelet derived growth factor
TNF‐α: Tumor Necrose Factor Alpha
VEGF: vascular endothelial growth factor

## INTRODUCTION

Tendinopathy is a pathological condition defined by pain, edema, and functional impairment, with a multifactorial etiology, involving factors such as repetitive overload and oxidative stress, which may result in degeneration of the tendon matrix and cellular dysfunction.^1^ The calcaneal tendon is among the most commonly affected sites, with a lifetime incidence of up to 52% in endurance runners.^2^ However, despite its strong association with physical activity, up to 31% of cases are reported in sedentary individuals.^3^ Tendinopathy negatively impacts both quality of life and productivity, with 25%–60% of patients experiencing symptoms for 5–10 years.^2^

Intrinsic factors contributing to the development of tendinopathy include altered biomechanics and systemic conditions, whereas extrinsic factors involve mechanical overload and training errors. Histologically, tendinopathy is characterized by an increased number of tenocytes, collagen disorganization, and neovascularization.^4^ In addition to tendon alterations, adjacent muscles, such as the gastrocnemius, are also affected due to the inflammatory process and disuse.^5^ The tendon functions as a mechanical bridge, facilitating muscle contraction and ensuring adequate functional adaptation.^6^ The tendon–muscle relationship has been demonstrated by a significant association observed between gluteus medius and minimus tendinopathy and muscle hypotrophy.^7^

Consequently, multiple rehabilitation strategies have been recommended for patients with tendinopathy. Although these approaches act through different mechanisms, their primary goals are to reduce symptoms—particularly pain—promote tendon healing, and improve patient function.^8^

Advanced platelet‐rich fibrin (A‐PRF) injection has emerged as a treatment for tendinopathy, promoting tissue regeneration by the sustained growth factors release such as TGF‐β and FGF, which stimulate fibroblasts and enhance extracellular matrix synthesis.^9^ Compared to PRP and standard PRF, A‐PRF releases higher amounts of growth factors over time, thereby supporting tendon repair.^10^ Its regenerative potential may accelerate healing, reduce inflammation, and improve functional outcomes.^11^

Photobiomodulation (PBM) is a non‐invasive therapy that alleviates pain and accelerates healing, making it useful in the treatment of tendinopathies. PBM stimulates cellular and biochemical processes, thereby promoting tissue regeneration. In tendons, it enhances fibroblast proliferation and collagen synthesis, leading to increased tensile strength.^12^ Studies have shown that PBM can modulate multiple mechanisms related to cell growth and proliferation, activate growth factors, and improve tissue regeneration, demonstrating benefits both in vitro and in animal models.^13^, ^14^

The literature has already reported muscular alterations resulting from tendinopathy; however, it remains uncertain whether targeted treatments for this condition are capable of reversing these changes. In this context, it is essential to investigate therapeutic interventions that address not only the tendon but also the associated muscular consequences. Therefore, studies comparing the effects of photobiomodulation (PBM) and advanced platelet‐rich fibrin (A‐PRF) on gastrocnemius muscle alterations induced by calcaneal tendinopathy are warranted. Accordingly, the present study aims to evaluate and compare the efficacy of PBM and A‐PRF in the treatment of these muscle sequelae secondary to tendinopathy.

## MATERIALS AND METHODS

This is a longitudinal experimental study that was approved by the Ethics Committee for the Use of Animals at the State University of Western Paraná, Cascavel Campus, under protocol number 12–23.

Seventy‐five adult Wistar rats, aged 9 ± 1 weeks and weighing an average of 274.77 ± 5.96 g, were used. They were housed in polypropylene cages in an air‐conditioned environment (22 ± 2°C) under a 12‐hour light/dark cycle. Throughout the experiment, they had free access to pelleted feed and water. The animals underwent a seven‐day acclimatization period, followed by 7 days of adaptation to the functional test. After this period, they were randomly assigned to the following experimental groups, with 15 animals in each group:

- Control group (CON): The animals did not receive any procedures.
- Lesion group (LES): Animals were subjected to calcaneal tendon injury and received no treatment.
- Photobiomodulation group (PBM): The animals were injured and treated with PBM.
- Advanced platelet‐rich fibrin group (A‐PRF): Animals were injured and treated with advanced platelet‐rich fibrin injection.
- Advanced platelet‐rich fibrin + photobiomodulation (A‐PRF + PBM) group: The animals were injured and treated with A‐PRF and PBM.

### Induction of tendinopathy

Animal cruelty standards have been adopted, as required by international standards. To induce tendinopathy, the animals were anesthetized with ketamine hydrochloride (75 mg/kg) and xylazine hydrochloride (15 mg/kg). Subsequently, the calcaneal tendon was subjected to transverse compression for 2 min using the first tooth of the Halstead forceps rack.^15^ The day of this procedure was called D0.

### Therapeutic protocols

#### Treatment A‐PRF

A 1.5 mL blood sample was obtained by cardiac puncture 16 from each animal in the corresponding group, using a 22G hypodermic needle. The blood samples were transferred to 2 mL Eppendorf tubes and immediately centrifuged at 3000 rpm (approximately 400G) for 10 min. After centrifugation, the product showed A‐PRF in the middle of the test tube. The A‐PRF was immediately removed from the tube with a 22G hypodermic needle for application to the animal's tendon, in just one application.^16^

#### Photobiomodulation (PBM) treatment

Low‐level laser equipment was used in the red spectrum (660 nm) in continuous radiation on the target; the other parameters are shown in Table 1. The applicator was placed at a 90° angle at three different points with an exposure duration of 28 s on the distal, medial, and proximal portions of the calcaneal tendon. To carry out the procedure, the animals were restrained manually.

**TABLE 1:** Photobiomodulation parameters.

Wavelength	660 nm
Power	30 mW
Spot	0.28 cm^2^
Irradiance	107.14 mW/cm^2^
Fluence	3 J/cm^2^
Energy (total)	2.52 J
Time (total)	84 s

### Functional evaluation, euthanasia and histomorphometric analysis

Functional data were collected immediately before induction on day 0 (D0). Subsequent assessments were performed every 7 days, on days 7, 14, and 21 (D7, D14, and D21).

Grip strength assessment: Grip strength was measured using a transducer (Insight®). The animal's forelimbs were manually restrained, along with the left hind limb, to prevent interference with the test. The animal was then positioned so that the right hind limb gripped the railing and was gradually pulled posteriorly by the trunk until the animal released its grip.^17^ The device recorded the force exerted by the animal to maintain the grip in grams‐force. The average of three measurements was used for analysis.

At the end of the treatment period, the animals were euthanized by intraperitoneal administration of an overdose of ketamine (180 mg/kg) and xylazine (30 mg/kg).^18^ Euthanasia was performed on days 7, 14, and 21 (D7, D14, and D21) for the animals in each group. The gastrocnemius muscle of the right hind limb was then collected. The muscle was carefully dissected, measured using a manual caliper, weighed on an analytical scale (Bel®), and sectioned into fragments for further analysis.

#### Histological procedures

Medial distal fragments of the lateral head of the gastrocnemius muscle were fixed in Metacarn solution (70% methanol, 20% chloroform, and 10% glacial acetic acid) for 24 h and subsequently stored in 70% ethanol. For slide preparation, the samples were dehydrated through a graded alcohol series, cleared in n‐butyl alcohol, and embedded in paraffin. The muscle was then sectioned using a Leipzig microtome into 7 μm‐thick cross sections. Histological slides were prepared and stained with Hematoxylin and Eosin (HE) and Picrosirius Red (PSR).

#### Histomorphometric analysis of the muscle fibers

After slide preparation, the samples were stained with hematoxylin and eosin (HE). For evaluation of the muscle tissue, an adapted pathological index for muscle tissue was applied. This index scored lesions on a scale from 0 to 6, where 0 = none, 2 = minimal, 4 = moderate, and 6 = severe, according to the extent of tissue involvement.^19^ For both general and quantitative analysis, the slides were examined under a 40× objective and photomicrographed at the same magnification. For quantitative assessment, 10 fields per animal were analyzed to measure the area, largest and smallest diameters of muscle fibers, total number of muscle fibers, nuclei, and capillaries, as well as the nucleus‐to‐fiber and capillary‐to‐fiber ratios. All muscle fibers, nuclei, and capillaries present in the complete photomicrograph were included in the analysis.

#### Histomorphometric analysis of connective tissue

After slide preparation, the samples were stained with Picrosirius Red. For analysis, 10 fields per animal were photomicrographed using a 20× objective. Connective tissue was quantified and differentiated based on the percentage of pixels counted in each image.^20^

To analyze NMJs, distal lateral fragments of the medial head of the gastrocnemius muscle were fixed in Karnovsky solution. The fragments were longitudinally sectioned into multiple slices using a stainless steel blade, and the sections were subjected to a non‐specific esterase reaction. For morphometric analysis, the area, largest diameter, and smallest diameter of 100 NMJs per animal were measured in microscopic images captured under a 20× objective.^21^

### Statistical analysis

Data are presented as mean ± standard deviation. The sample size was calculated using an effect size of 0.53, an alpha level of 5%, and a statistical power of 95%, using the G*Power software. Inferential analysis was performed using the Generalized Linear Model, followed by the LSD post hoc test. A significance level of 5% was adopted. All analyses were conducted using SPSS version 20.0. Effect sizes were calculated using Hedges' g and interpreted as follows: insignificant <0.19; small 0.20–0.49; medium 0.50–0.79; large 0.80–1.29; very large >1.30.^22^

## RESULTS

A total of 59 animals were euthanized at the end of the experimental period. Sixteen animals were excluded due to mortality associated with anesthesia and cardiac puncture procedures. The distribution of losses was as follows: three animals in the LES group, two in the PBM group, five in the A‐PRF group, and six in the A‐PRF + PBM group.

### Body mass of the animals

LES showed a reduction in body mass compared to CON, which was significantly lower (*p* < 0.001). The same was observed in the PBM in relation to the CON (*p* < 0.001), in the A‐PRF (*p* < 0.001) and in the A‐PRF + PBM (*p* = 0.01). There was no significant difference in the length and width of the gastrocnemius muscle between the groups. However, when the mass of the gastrocnemius muscle was compared, LES showed a reduction compared to CON (*p* < 0.001). The same was observed in PBM in relation to CON (*p* = 0.00), in A‐PRF (*p* < 0.001) and in A‐PRF + PBM (*p* = 0.01). The data are shown in Table 2.

**TABLE 2:** Anthropometric measurements of animals between 10 and 13 weeks of age.

Groups	Animal weight (g)	Muscle weight (mg)	Muscle length (mm)	Muscle width (mm)
CON	301.40 ± 6.54^a^	1.4112 ± 0.913^a^	24.47 ± 0.601^a^	19.13 ± 0.678^a^
LES	270.67 ± 5.87^b^	1.3266 ± 0.864^b^	23.91 ± 0.702^a^	18.27 ± 0.792^a^
PBM	269.80 ± 5.86^b^	1.3122 ± 0.849^b^	24.54 ± 0.646^a^	18.77 ± 0.729^a^
A‐PRF	258.66 ± 5.61^b^	1.2476 ± 0.776^b^	23.60 ± 0.736^a^	17.30 ± 0.831^a^
A‐PRF + PBM	273.33 ± 5.93^b^	1.3570 ± 0.749^b^	24.44 ± 0.776^a^	19.33 ± 0.876^a^

*Note*: Values followed by the same letter do not differ significantly from each other using the Generalized Linear Model test.

Abbreviations: A‐PRF + PBM, advanced platelet‐rich fibrin plus photobiomodulation; A‐PRF, advanced platelet‐rich fibrin; CON, control; LES, lesion; PBM, photobiomodulation.

### Functional test

**FIGURE 1:**
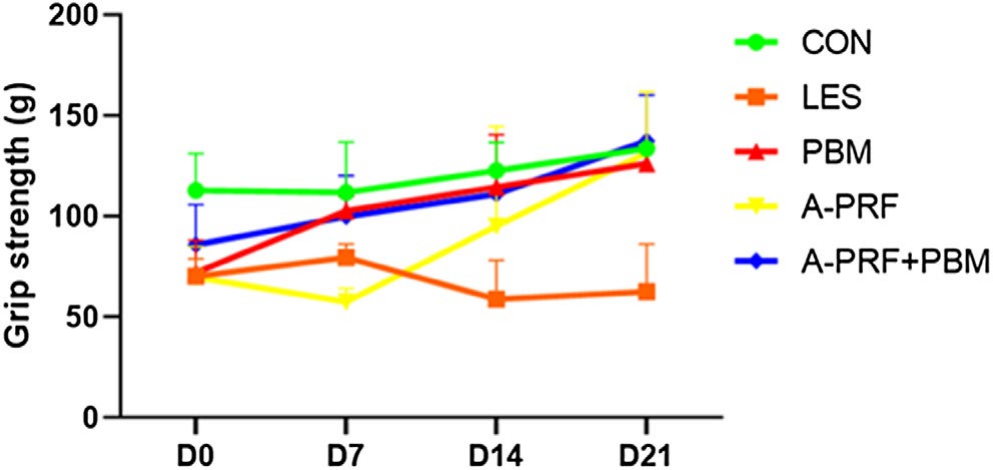
Graphical presentation of the grip strength values obtained from the different groups at the time of assessment. A‐PRF + PBM, advanced platelet‐rich fibrin plus photobiomodulation; A‐PRF, advanced platelet‐rich fibrin; CON, control; LES, lesion; PBM, photobiomodulation.

Regarding the functional grip strength test, the CON group maintained higher average muscle strength than the other groups during the first 14 days. By day 21, however, the CON group exhibited muscle strength similar to that of the other groups, except for the LES group. The LES group maintained muscle strength during the first week but showed a decline from the second week onward, finishing the experiment with the lowest average muscle strength among all groups. The PBM group demonstrated a gradual increase in muscle strength over the experimental period. The A‐PRF group showed a decrease in muscle strength during the first week but recovered in subsequent weeks. The A‐PRF + PBM group exhibited a gradual increase in muscle strength over time. These results are illustrated in Figure 1, with effect sizes presented in Table 3.

**TABLE 3:** Presentation of effect sizes compared to day zero (DO) within each group.

Groups	Assessment day	Assessment day	Effect size
CON	D0	D7	−0.051
		D14	0.567
		D21	1.257
LES	D0	D7	1.126
		D14	−0.998
		D21	−0.599
PBM	D0	D7	2.079
		D14	2.746
		D21	3.701
A‐PRF	D0	D7	−0.884
		D14	1.042
		D21	3.334
A‐PRF + PBM	D0	D7	0.701
		D14	1.375
		D21	2.552

*Note*: Effect sizes calculated by Hedges' g with the following interpretation: insignificant <0.19; small 0.20–0.49; medium 0.50–0.79; large 0.80–1.29; very large >1.30.

Abbreviations: A‐PRF + PBM, advanced platelet‐rich fibrin plus photobiomodulation; A‐PRF, advanced platelet‐rich fibrin; LES, lesion; PBM, photobiomodulation.

### Morphological and histomorphometric analysis of muscle tissue

**FIGURE 2:**
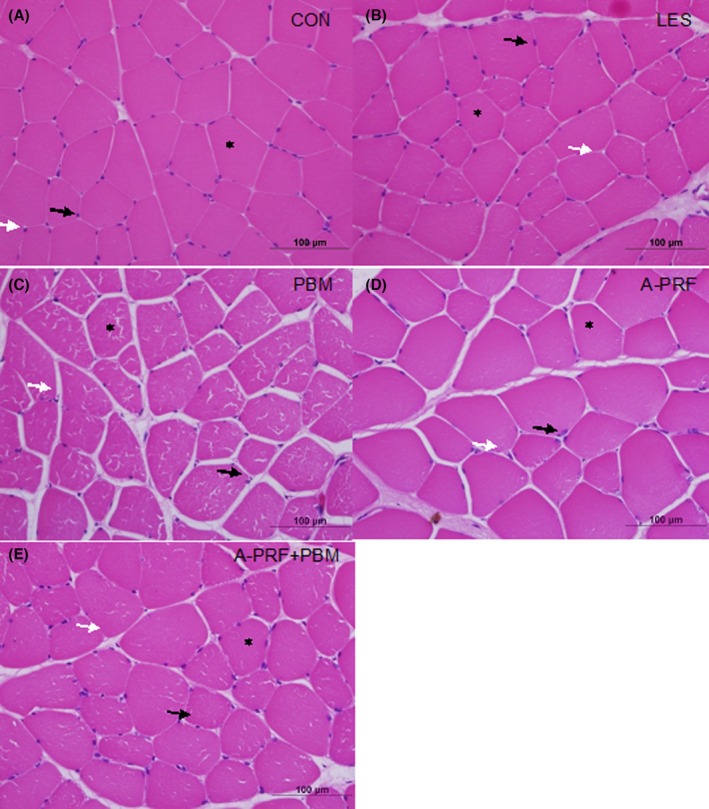
Photomicrographs of the gastrocnemius muscle of rats aged between 10 and 13 weeks. Cross section of muscle fiber stained with hematoxylin and eosin at 40× magnification (A–E). A‐PRF + PBM, advanced platelet‐rich fibrin plus photobiomodulation; A‐PRF, advanced platelet‐rich fibrin; CON, control; LES, lesion; PBM, photobiomodulation. (A–E) polygonal muscle fiber (asterisk), peripheral nucleus (black arrow), and blood capillary (white arrow).

No significant differences were observed in the gastrocnemius muscle between groups according to the pathological index analysis, with all alterations scored as 0 (Figure 2). Similarly, analysis of muscle fiber area, largest and smallest diameters, total number of muscle fibers, nuclei, capillaries, and the nucleus‐to‐fiber ratio revealed no significant differences between groups. However, for the capillary‐to‐fiber ratio, the CON and PBM groups were significantly higher than the A‐PRF group (*p* = 0.01 for both comparisons) (Figure 2).

### Histomorphometric analysis of connective tissue

**FIGURE 3:**
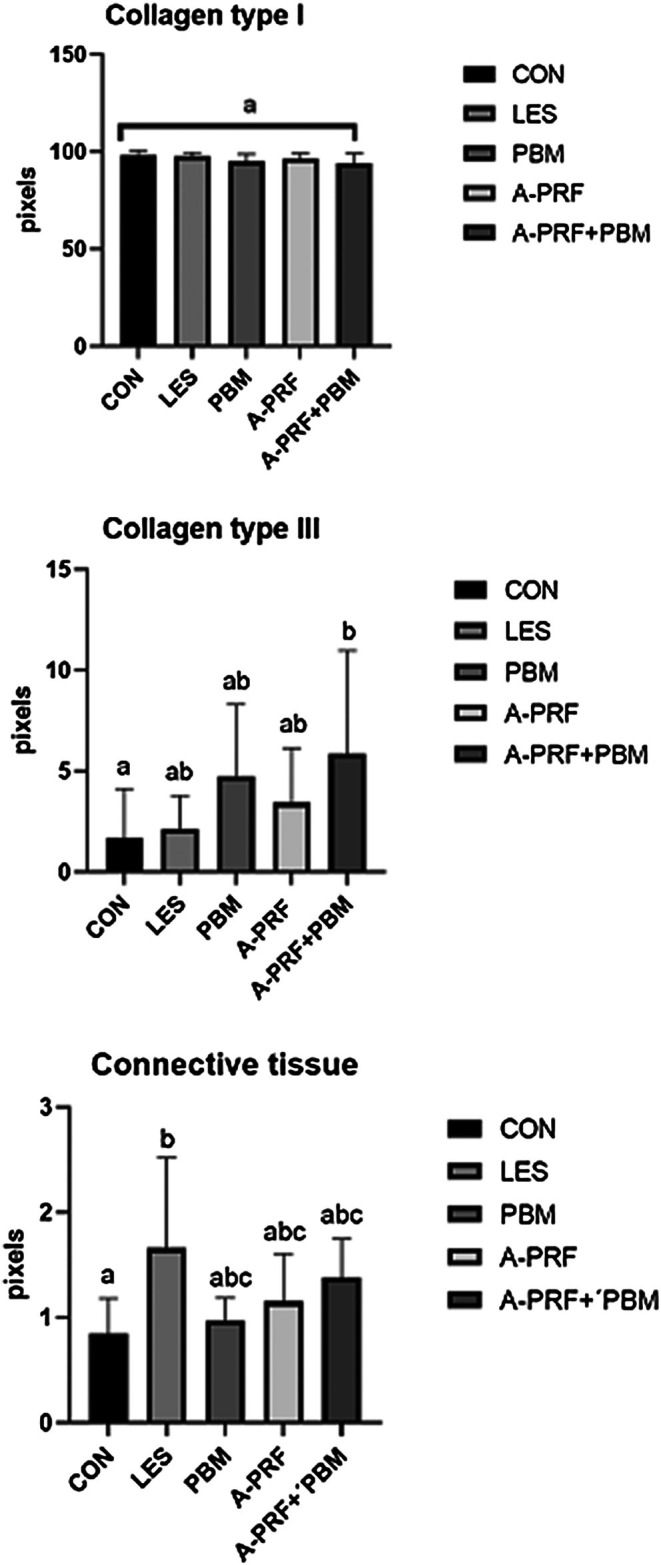
Graphical presentation of the values for the observed values in pixels, of type I collagen, type III collagen, and connective tissue, according to the groups. When there are similar letters, there is no statistical difference. A‐PRF + PBM, advanced platelet‐rich fibrin plus photobiomodulation; A‐PRF, advanced platelet‐rich fibrin; CON, control; LES, lesion; PBM, photobiomodulation.

**FIGURE 4:**
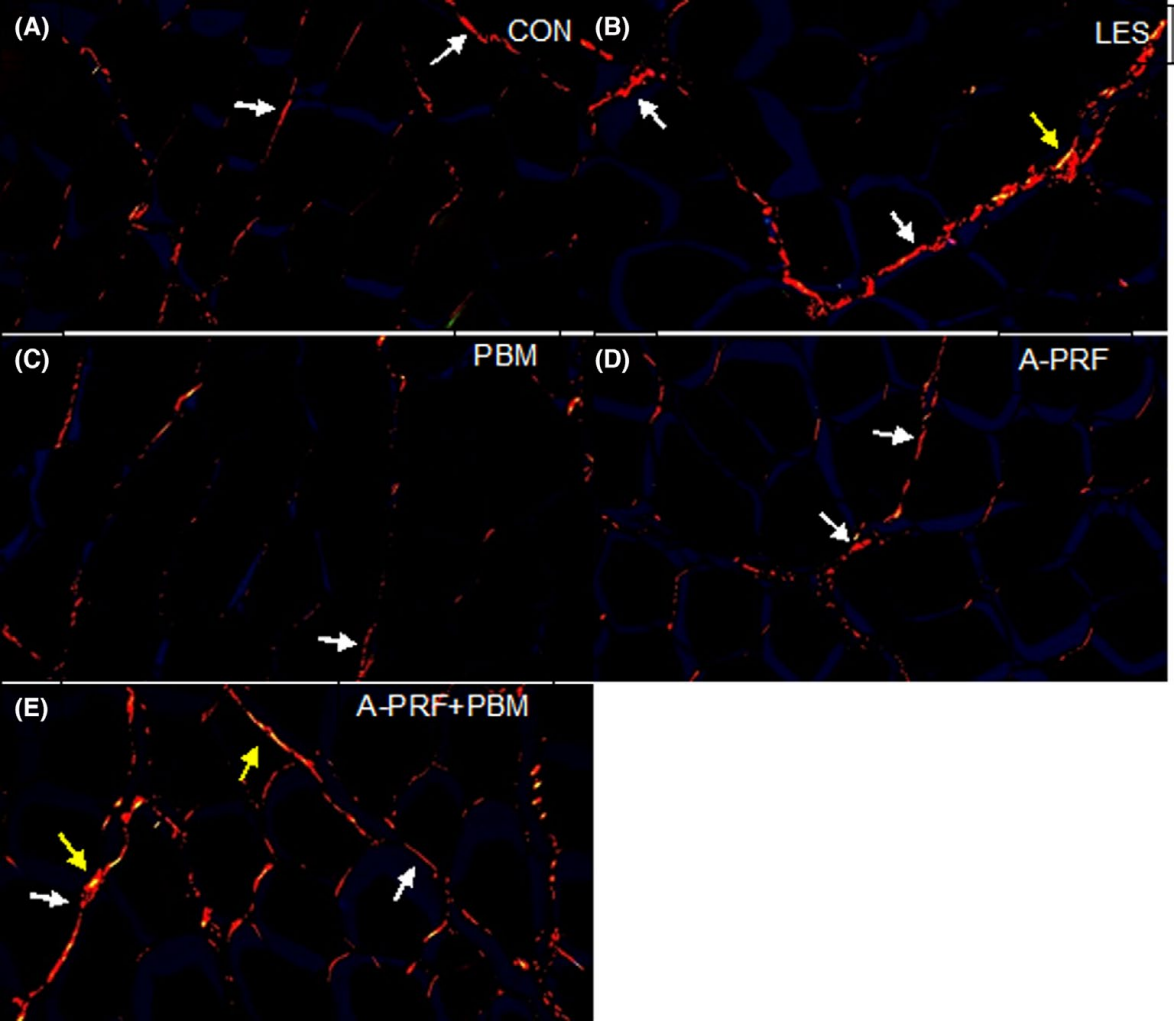
Photomicrographs of the gastrocnemius muscle of rats aged between 10 and 13 weeks. Transverse section after staining in Picrosirius red at 20× magnification (A–E). A‐PRF + PBM, advanced platelet‐rich fibrin plus photobiomodulation; A‐PRF, advanced platelet‐rich fibrin; CON, control; LES, lesion; PBM, photobiomodulation. (A–E) white arrow (collagen type I), yellow arrow (collagen type III).

Analysis of type I collagen revealed no significant differences between groups. However, for type III collagen, the A‐PRF + PBM group showed a significant difference in the gastrocnemius muscle compared to the CON group (*p* = 0.03). In the analysis of connective tissue, the LES group differed significantly from the CON (*p* < 0.001) and PBM (*p* = 0.01) groups, while the A‐PRF + PBM group showed a significant difference compared to CON (*p* = 0.02) (Figures 3 and 4).

### Histomorphometric analysis of Neuromuscular Junctions

**FIGURE 5:**
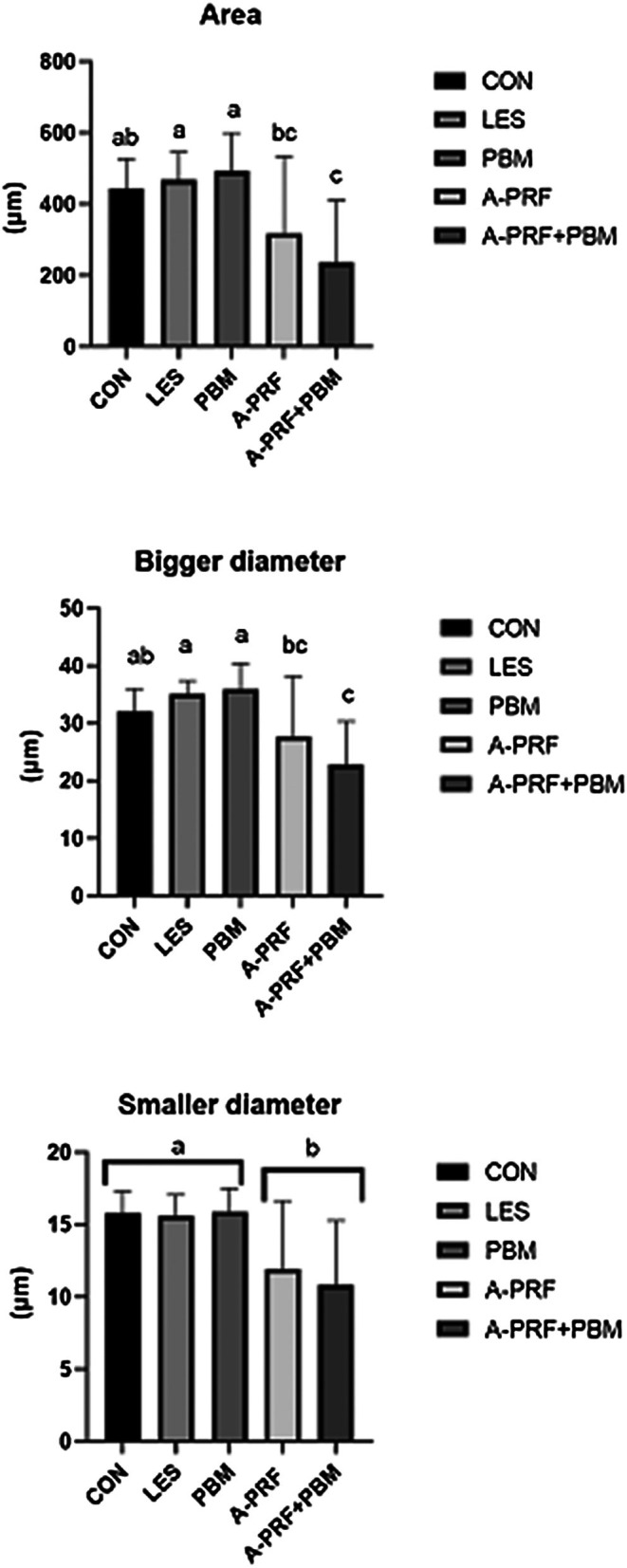
Graphical presentation of the values for the observed values in μm, the area, largest and smallest diameter of the neuromuscular junctions. When there are similar letters, there is no statistical difference. A‐PRF + PBM, advanced platelet‐rich fibrin plus photobiomodulation; A‐PRF, advanced platelet‐rich fibrin; CON, control; LES, lesion; PBM, photobiomodulation.

**FIGURE 6:**
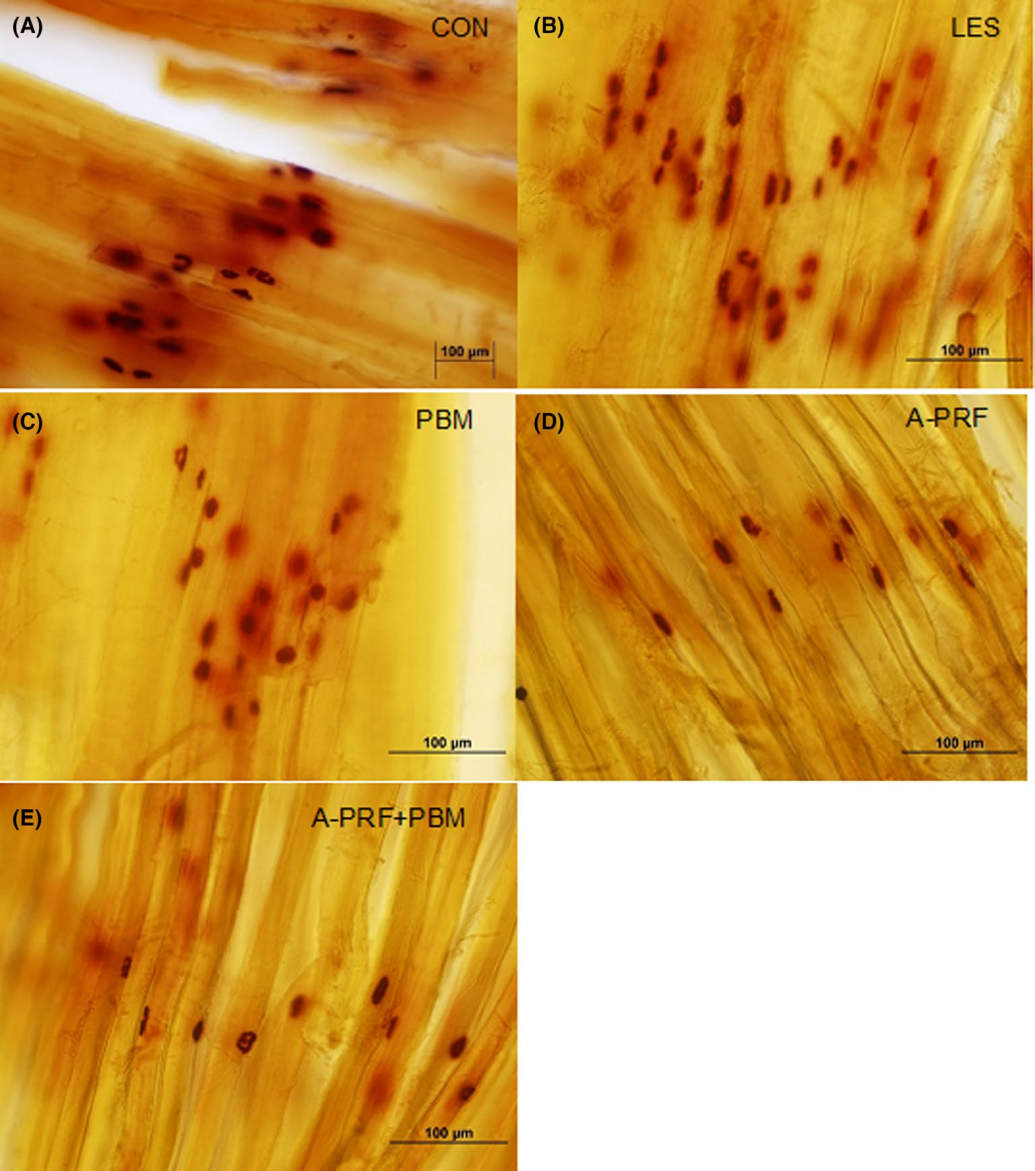
Photomicrographs of the gastrocnemius muscle myoneural junction of rats aged between 10 and 13 weeks. Longitudinal section after nonspecific esterase reaction at 20× magnification (A–E). A‐PRF + PBM, advanced platelet‐rich fibrin plus photobiomodulation; A‐PRF, advanced platelet‐rich fibrin; CON, control; LES, lesion; PBM, photobiomodulation.

A‐PRF + PBM had a smaller area than CON (*p* = 0.01). The NMJ area of the LES was greater in relation to the A‐PRF (*p* = 0.05) and the A‐PRF + PBM (*p* < 0.001). PBM had a larger NMJ area than A‐PRF (*p* = 0.02) and A‐PRF + PBM (*p* < 0.001). A‐PRF + PBM showed a larger diameter of the smaller NMJs than CON (*p* = 0.01). LES and PBM were also different in relation to the A‐PRF (*p* = 0.03 and *p* = 0.02) and A‐PRF + PBM (*p* < 0.001 and *p* < 0.001) groups. The smallest diameter of the NMJs had a higher average in the CON, LES, and PBM groups. These showed a significant difference in relation to the groups that used A‐PRF, which were A‐PRF (*p* = 0.02; *p* = 0.03 and *p* = 0.02) and A‐PRF + PBM (*p* = 0.00; *p* = 0.00 and *p* = 0.00) (Figures 5 and 6).

## DISCUSSION

This study investigated the effects of calcaneal tendon injury treated with PBM and A‐PRF on the gastrocnemius muscle of Wistar rats. To induce the injury, an adaptation of a technique involving direct compression through the skin was employed. Although some authors have previously used this injury model,^15^, ^23^ neither study evaluated the periarticular muscles to determine the full extent of the injury.

Effect size analysis indicated that the LES group experienced a reduction in muscle strength over the course of the experiment, possibly due to arthrogenic muscle inhibition, a protective mechanism triggered by periarticular muscle damage.^24^ This mechanism limits motoneuron recruitment and muscle activation by stimulating inhibitory interneurons that synapse with motor neurons.^25^ There is an association between soleus arthrogenic inhibition and ankle disability in patients with acute ankle sprain.^26^

It was also observed that the LES group exhibited an increase in connective tissue compared to the PBM and CON groups. This increase likely reflects a common physiological response to muscle injury, in which infiltration of inflammatory cells and formation of fibrous tissue occur as part of the healing process to repair the damaged muscle.^27^

Red‐spectrum photobiomodulation is widely used in the treatment of superficial and apparent lesions due to its effectiveness in stimulating fibroblasts.^28^ Accordingly, a wavelength of 660 nm was selected, as the calcaneal tendon is located superficially beneath the skin, and fibroblast stimulation is essential for promoting its repair.^29^

Effect size analysis indicated that the groups treated with PBM exhibited a progressive increase in muscle strength. The literature suggests that PBM is effective in muscle recovery, reducing fatigue and enhancing physical performance.^12^ The proposed mechanism involves light absorption by the enzyme cytochrome c oxidase, which stimulates the electron transport chain, increases ATP production, and provides additional energy to muscle cells during exercise.^30^ Furthermore, PBM can promote phosphocreatine resynthesis, enhancing mitochondrial activity and restoring the energy required for muscle contraction.^31^ It is also suggested that PBM aids in the removal and oxidation of lactic acid, a co‐product of anaerobic metabolism that induces fatigue, preventing or delaying a decrease in performance.^32^

In this study, PBM maintained the capillary‐to‐fiber ratio at levels similar to those of the CON group, thereby reducing capillary loss. In contrast, the A‐PRF group showed a reduction in this measure compared to PBM and CON. This finding is consistent with Nakano et al.,^33^ who reported a higher capillary‐to‐myofiber ratio in muscles treated with PBM following the induction of muscle atrophy, demonstrating that PBM promotes muscle angiogenesis.

Although no significant differences were observed in the muscle strength evaluations, the CON group exhibited a gradual increase, which may be explained by the concept of ergogenic aids that enhance physiological adaptations, including improved aerobic capacity, strength, and recovery, thereby increasing the efficiency of energy utilization.^33^ Such adaptations can be achieved through specific strategies and training. For example, a study demonstrated that cyclists acclimatized to heat conditions obtained ergogenic benefits when performing in cold environments.^34^

The selection of A‐PRF is based on its biological properties, including the sustained release of growth factors such as IGF‐1, PDGF, VEGF, FGF, and EGF, as well as fibrin matrix proteins, surpassing the effects of PRP and conventional PRF. This prolonged release promotes more efficient tendon regeneration, particularly in chronic injuries.^11^ Additionally, A‐PRF forms a three‐dimensional fibrin matrix that serves as a scaffold for the migration and activity of tendon repair cells.^10^

The A‐PRF group exhibited a reduction in muscle strength during the first week, followed by recovery in subsequent weeks. This initial decrease may be attributed to a temporary inflammatory response at the treated site, which is common in the early days and can cause muscle pain and discomfort, thereby affecting strength.^35^, ^36^ Following this phase, A‐PRF promotes muscle regeneration, leading to faster recovery and, over the long term, enhanced muscle strength.^37^, ^38^

Type III collagen is one of the first forms of collagen deposited in damaged tissues. It plays a critical role during the proliferative phase, predominating and facilitating tissue regeneration by forming a less dense network that supports cell migration, new matrix deposition, and angiogenesis. Therefore, an increase in type III collagen in injured muscle is an essential mechanism for promoting recovery and reorganization of damaged tissue.^39^, ^40^ In this study, only the combined A‐PRF + PBM treatment resulted in an increase in type III collagen, whereas the individual treatments showed no significant differences between groups. This effect is likely due to the biomodulatory action of the combined therapies, which regulate cellular and biochemical processes to control inflammation, promote regeneration, and relieve pain. These effects include a reduction in proinflammatory cytokines, such as TNF‐α, IL‐1β, and IL‐6, and an increase in IL‐10, an anti‐inflammatory cytokine that favors tissue repair.^41^ These findings are consistent with another study,^42^ in which combined fibrin sealant and PBM therapy resulted in superior bone graft healing compared to either treatment alone.

As healing progresses, type III collagen is gradually replaced by type I collagen, which is stronger and contributes to the restoration of muscle strength and function.^43^ In this study, no significant increase in type I collagen was observed between groups. This is likely because the transition to type I collagen occurs gradually, and a longer experimental period may be required to detect its increased presence. This slow replacement process has been reported previously, where a notable increase in type I collagen was observed only after 30 days post‐injury.^43^

Tendon healing typically progresses through an inflammatory phase (days), a repair phase (weeks), and a remodeling phase (months).^44^ A previous study^45^ demonstrated, through histological analysis, that PRF implantation initiates the tendon repair process, characterized by increased proliferation and differentiation of tenocytes. In the present study, the A‐PRF and A‐PRF + PBM groups exhibited neuromuscular junctions (NMJs) with smaller areas, larger maximum diameters, and smaller minimum diameters compared to groups not treated with A‐PRF. This finding can be explained by the fact that, during the repair phase, mechanical stress on the tendon and adjacent tissues can alter NMJ morphology and functionality, promoting adaptations such as increases or reductions in functional area.^45^

A limitation of this study is the absence of analysis of the molecular characteristics of inflammation using inflammatory biomarkers. For future research, we recommend performing immunoenzymatic assays to evaluate both pro‐ and anti‐inflammatory cytokines, as well as employing an injury model with a surgical incision to more accurately assess the extent of muscle damage.

## CONCLUSION

The combined therapies resulted in greater muscle tissue repair, as evidenced by the increase in type III collagen, compared to the individual treatments. Photobiomodulation therapy, however, was effective in increasing the capillary‐to‐fiber ratio, an effect not observed with A‐PRF alone. In the case of A‐PRF therapy, a reduction in NMJ size was observed, which was not seen in the other groups. Importantly, both treatments were able to restore muscle strength by the end of the experiment.

## FUNDING INFORMATION

Thanks to Conselho Nacional de Desenvolvimento Científico e Tecnológico (CNPq) and Fundação Coordenação de Aperfeiçoamento de Pessoal de Nível Superior (CAPES).

## ETHICS STATEMENT

The project was approved by the Ethics Committee for the Use of Animals at the Universidade Estadual do Oeste do Paraná, Cascavel Campus, under opinion No. 12‐23.

## Data Availability

The data that support the findings of this study are available from the corresponding author upon reasonable request.
